# Editorial: NLR-Protein Functions in Immunity

**DOI:** 10.3389/fimmu.2015.00306

**Published:** 2015-06-11

**Authors:** Jörg H. Fritz, Thomas A. Kufer

**Affiliations:** ^1^Department of Microbiology and Immunology, Complex Traits Group, McGill University, Montreal, QC, Canada; ^2^Institute of Nutritional Medicine, Department of Immunology, University of Hohenheim, Stuttgart, Germany

**Keywords:** innate immunity, NLR, protein, microbes, inflammatory responses

Since Janeway (1) and Matzinger (2) put forward two distinct concepts of innate immune recognition, arguing that the driving force that initiates immune reponses is the recognition of microbial patterns or endogenous danger signals, respectively, we have acquired a tremendous wealth of knowledge of the protein families involved. Host-encoded pattern-recognition molecules (PRM) sense conserved microbial structures, referred to as microbe-associated molecular patterns (MAMP) or pathogen-associated molecular patterns (PAMP), as well as endogenous danger-associated molecular patterns (DAMP). Protein families of PRM include the well-described membrane-associated toll-like receptors (TLR) (3–5) and C-type lectins (6). Beside that, the host also evolved intracellular PRM that were identified only more recently (7, 8). One important class of such intracellular PRM is the family of NOD-like receptor (NLR) proteins (9).

In this research topic on “NLR-protein functions in immunity” leading experts in the field discuss various aspects of NLR biology. The proteins of the NLR family are evolutionary conserved molecules that in plants and mammals have been implicated in innate immune sensing of microbes and infection-associated physiological changes, contributing to immune protection of the challenged host organism through the instruction of inflammatory responses, antimicrobial defense, and adaptive immunity.

Plant NLR, in contrast to mammalian NLR, recognize pathogen-derived effector molecules or the activity of these in the cytosol and can act as transcriptional regulators in the nucleus. Notably, the function of most of these proteins is conserved in phylogenetically distant species. Jacob and co-workers present current concepts on the evolution and function of NLR in plants providing an insightful comparison of the repertoire of NLR and NLR-like proteins in different plant species (10). The wiring of plant NLR to signal transduction processes, their molecular activation, and the role of sub-cellular localization are covered by a review by Qi and Innes (11).

To date, our structural understanding of the mechanisms underlying activation and signaling by NLR is hampered by the intrinsic difficulty to obtain recombinant proteins suitable for structural assessment. However, functional studies and in particular evolutionary perspectives allow the acquisition of novel insights into these mechanisms. Monie and colleagues provide new insights by an evolutionary analysis of the NLR proteins Nod1 and Nod2, defining the nature of the interaction surfaces with their ligands and downstream adaptors (12).

Since the first report demonstrating the involvement of NLR in sensing bacterial components (13), their contributions to the control of infection and their impact on immune regulation is becoming increasingly understood. Opitz and co-workers summarize our understanding of the function of NLR in infectious lung diseases (14). In addition, Rosenstiel and Lipinski (15) and Flavell and colleagues (16) detail the roles of NLR in sensing intestinal bacteria in regulating intestinal immune homeostasis at steady state and during infectious challenge. Ferrero and co-workers describe how NLR drive immunity toward extracellular bacteria by recognition of MAMP in released bacterial outer-membrane vesicles (17), while Olivier and colleagues discuss the role of NLR in sensing malarial pigment hemozoin (18). Recently, the role of xenophagy in anti-bacterial host-defense is becoming increasingly evident and NLR proteins have been shown to be involved in triggering this cellular event. The molecular details of how NLR target the autophagy pathway are discussed by Carneiro and Travassos (19).

The functional interplay between NLR and regulatory proteins is a research area with many open questions. Le and Harton discuss how a family of Pyrin- and CARD-only proteins interact with NLR to regulate signaling (20).

It is well accepted that activation of PRM is pivotal to trigger adaptive immunity. The review by Eisenbarth (21) details the impact of NLR proteins in shaping antigen-specific immune responses. The role of the NLR member CIITA, acting as a master regulator for MHC expression, is long known. Very recently, several laboratories demonstrated that NLRC5 exerts a key role in MHC expression as well. Kufer and colleagues provide an overview of the current progress in understanding the function of NLRC5 (22).

The important contribution of NLR proteins to immune homeostasis is well underscored by the findings that polymorphisms in their human genes that are linked to disease. Saleh and colleagues provide an overview of known associations of NLR and disease (23), and Kanneganti and Lupfer discuss open questions of NLR biology (24). Moreover, Stehlik and co-workers detail the role of mutations in PYD-containing NLR in disease, which are important for inflammasome formation that drives Il-1β and IL-18 release (25). Finally, the increasingly recognized role of NLR in carcinogenesis is reviewed by Sutterwala and colleagues (26).

Although most mammalian NLR proteins contribute to immunity, some members of this family show restricted expression in the germ line and are associated with developmental processes. This often-neglected role of NLR is illustrated by the consequences of polymorphisms in NLRP7, resulting in embryonic malformations that are reviewed and discussed by Slim and Wallace (27).

The NLR and NLR-like molecules in mammals and plants, respectively, represent a very interesting protein family with diverse functions expanding beyond immune regulation. Particularly in plants, we witnessed many important advances for our understanding of the functions of NLR in cell autonomous pathogen recognition and subsequent signaling. Although first described about 20 years ago, our understanding of the biology of many NLR members in mammals is still fragmentary. Much progress has been made regarding the characterization of the biology of Nod1, Nod2, NLRC4, and NLRP3. In contrast, other NLR have still not been experimentally assessed at all. The collection of articles presented here aims to give an overview of our current understanding of NLR functions and highlight open questions. Utilizing more powerful genetics and advanced cell biology tools will enable us to address controversies in the field and help to further our understanding of the biology of this important protein family.

## Conflict of Interest Statement

The authors declare that the research was conducted in the absence of any commercial or financial relationships that could be construed as a potential conflict of interest.
